# Recent Developments and Applications of Terahertz Spectroscopy in Food Analysis

**DOI:** 10.3390/bios15100677

**Published:** 2025-10-08

**Authors:** Pengpeng Yu, Chaoping Shen, Wenhui Zhu, Wenya Zhang, Junhui Cheng, Jinxiu Song

**Affiliations:** 1 School of Agricultural Engineering, Jiangsu University, Zhenjiang 212013, China; 2 Aviation Engineerng Institute, Jiangsu Aviation Technical College, Zhenjiang 212134, China; 3 School of Energy and Power Engineering, Jiangsu University, Zhenjiang 212013, China; 4 Tianshui Huatian Technology Co., Ltd., Tianshui 741000, China; 5 College of Water Resources and Civil Engineering, China Agricultural University, Beijing 100083, China

**Keywords:** Terahertz spectroscopy, food safety, quality assessment, adulteration identification, component analysis

## Abstract

The terahertz waves are electromagnetic waves with frequencies ranging from 0.1 to 10 THz, exhibiting characteristics of both microwave and infrared, including fingerprint characteristics, coherence, and safety. Due to the weak interactions among most organic macromolecules in substances, the vibrational modes of molecular frameworks, as well as dipole rotation and vibration transitions, often correspond to the terahertz spectral region. Consequently, there has been growing interest in applying terahertz technology within the food industry. This review summarizes the fundamental principles of terahertz spectroscopy for substance detection and highlights recent advances and applications in food analysis. Key applications include harmful contaminant detection, component analysis, quality assessment, and adulteration identification. Additionally, this review discusses current challenges in applying terahertz spectroscopy to food analysis, such as strong water absorption, matrix interference, and the lack of comprehensive spectral databases. Finally, the paper outlines future prospects, including the development of lightweight and cost-effective terahertz sources and detectors for on-site analysis, as well as the integration of terahertz spectroscopy with other modern detection technologies to enhance analytical performance. This work aims to serve as a reference for further research and development of terahertz spectroscopy in the food sector.

## 1. Introduction

With the high-quality development of the economy, people’s living standards have been greatly improved; the role of agricultural products is no longer just to feed, and their quality has become the focus of attention [1,2]. However, various factors in the process of production, processing, transportation and sales of agricultural products can easily lead to adulteration of ingredients, excessive residues of harmful substances and other problems, which seriously damage the quality of agricultural products [3,4,5]. In order to ensure food safety and food quality, people have developed some traditional detection methods based on large instruments, such as chromatography, chromatography–mass spectrometry and capillary electrophoresis [6,7,8]. These methods have limitations, such as requiring specialized personnel to operate, cumbersome sample handling, and lossy detection of analytes [9,10]. Therefore, it is imperative to develop low-cost, simple, rapid, accurate and non-destructive detection and analysis methods.

In recent years, many emerging spectral technologies, such as Raman spectroscopy, terahertz spectroscopy, fluorescence spectroscopy, and near-infrared spectroscopy, have gradually attracted the attention of researchers due to their advantages of being fast, simple and non-destructive [11,12,13,14]. Compared with traditional radiation sources, terahertz radiation offers the benefits of strong penetration, transient and broadband properties, coherence, safety, and low energy [15,16]. However, Raman spectroscopy is often affected by interference from fluorescence background noise, and its spectral signal tends to be weak. The development of surface-enhanced Raman spectroscopy has overcome the challenge of weak signals, but it still faces issues related to poor stability and reproducibility. Fluorescence spectroscopy is prone to light bleaching, and fluorescence probes generally have high costs. The resolution of near-infrared spectroscopy is relatively low and is easily influenced by factors such as particle size and sample uniformity. In contrast, although terahertz spectroscopy is sensitive to water content, it can achieve deep, non-destructive detection within packaging and directly capture molecular conformational changes and weak interactions. This represents a unique advantage of terahertz spectroscopy compared to other spectroscopic techniques. The weak interaction between molecules has obvious characteristic absorption peaks in the terahertz band, and shows strong absorption and dispersion characteristics, which can be used as the “fingerprint” spectrum of the substance. Therefore, the terahertz frequency band contains rich biomolecular information, and many biomolecular vibration and rotation occur in the terahertz frequency band [17]. The core technical framework of terahertz spectroscopy, terahertz time-domain spectroscopy (THz-TDS), was first proposed by Professor Daniel Richard Grischkowsky and was applied to the study of the absorption characteristics of water vapor [18,19]. In recent years, with the rapid development of terahertz sources and detectors, terahertz spectroscopy has shown great application potential in the field of food analysis [20,21]. Terahertz spectroscopy is highly suitable for on-site processing analysis, such as dehydration, fermentation, freezing, storage and other production and preservation processes [22,23,24,25]. In addition, combining the stoichiometric method with the terahertz spectral information can further realize the quantitative and qualitative analysis of various macromolecular substances [26,27,28]. However, many substances have a limited response to the terahertz band, and to overcome this limitation, various metamaterials have been developed to significantly amplify the response of terahertz waves to target molecules [29]. Metamaterial is a kind of artificially designed material with periodic structure. Using the unique properties of metamaterials, researchers can design structures that provide precise control of electromagnetic fields and enable specific modulation of electromagnetic waves to achieve the desired function [30]. Terahertz metamaterials are labeled and fast optical sensors that take advantage of the advantages of the terahertz band and the unique electromagnetic properties of metamaterial devices. This allows them to achieve sensing capabilities that are more sensitive and efficient than other sensing technologies [31]. Combining with metamaterials, it is also possible to conduct quantitative analysis of harmful substances such as pesticide residues, antibiotics, and pathogenic bacteria in food [32,33,34,35].

**Figure 1:**
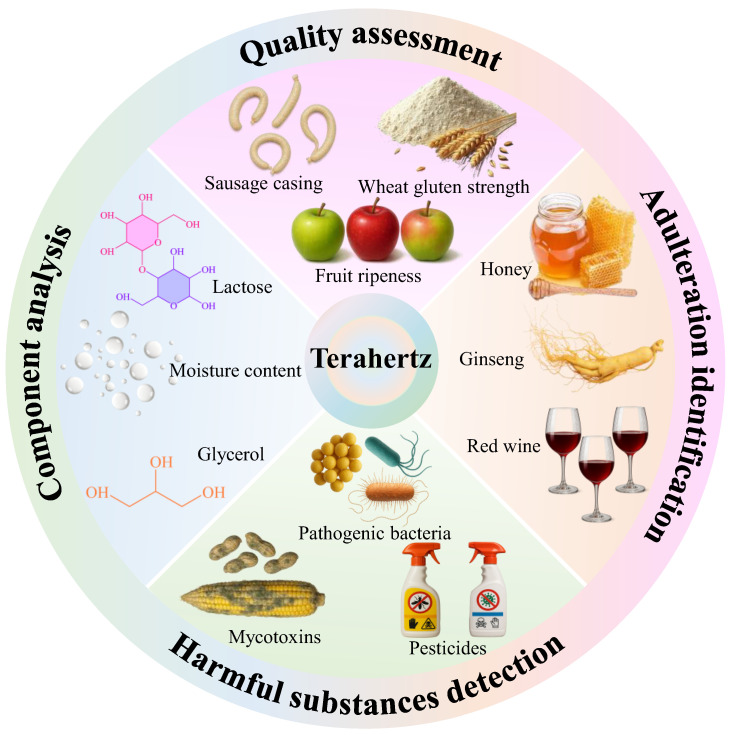
Schematic illustration of various application of terahertz technology in the food industry.

Given the current demands in agriculture and food engineering, we attempted to comprehensively understand the development and application status of terahertz spectroscopy technology in food quality assessment, adulteration identification, component analysis, and harmful substance detection (Figure 1). When combined with metamaterials, it can be used for the detection of pesticide residues, food additives, antibiotics, mycotoxins, and pathogenic bacteria. Finally, we summarized the main problems and technical difficulties currently existing in terahertz spectroscopy detection technology and looked forward to its future development prospects. This review will provide valuable insights into the future development of terahertz spectroscopy technology and its applications in various fields such as food safety, medical diagnosis, and environmental monitoring.

## 2. Principles of Terahertz Spectroscopy

### 2.1. Terahertz Time Domain Spectroscopy (THz-TDS) System

**Figure 2:**
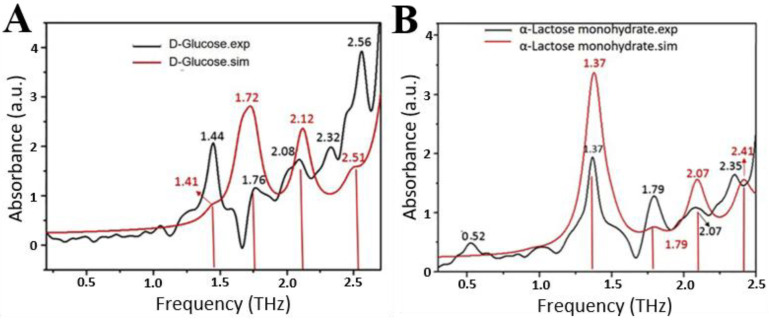
Experimental and simulated terahertz spectra of (**A**) D-glucose and (**B**) α-lactose. Adapted with permission from [39]. Copyright 2022 Elsevier.

Terahertz waves refer to electromagnetic waves with frequencies between 0.1 and 10 THz (1 THz = 10^12^ Hz), between microwave and infrared radiation in the electromagnetic spectrum [36,37]. Terahertz waves possess the characteristic of “molecular fingerprint spectra”. The low-frequency vibrations and rotational modes of many biochemical molecules, the phonon modes of crystals, the backbone vibration modes of large molecules, and the vibration modes caused by weak intermolecular interactions all correspond to the terahertz frequency range [38]. For example, Huang et al. measured the terahertz absorption spectra of D-glucose and α-lactose hydrates using the THz-TDS system and obtained their characteristic absorption peaks [39]. They also determined the corresponding assignments of the absorption peaks based on the simulation results. As shown in Figure 2A, the absorption peaks of D-glucose are located at 1.44, 1.76, 2.08, 2.32, and 2.56 THz. The characteristic peaks at 1.44, 1.76, 2.08, and 2.56 THz, respectively, belong to intermolecular interaction and collective vibration, the rotation of the six-membered ring and the wagging of –CHOH and –OH, the wagging of –OH and the rotation of –CHOH, the slight rotation of the six-membered ring and the wagging of –CHOH. The characteristic absorption peaks of α-lactose are located at 0.52, 1.37, 1.79, 2.07, and 2.35 THz. The peaks at 1.37, 1.79, 2.07, and 2.35 THz mainly originate from the collective vibration, collective translation of molecules, the translation of the six-membered ring and rotation of –CHOH and –COOH, rotation of –CHOH, –CH_2_OH and –OH. Furthermore, terahertz waves are highly penetrative and have low photon energy, meaning they do not cause ionization damage to biological molecules [40]. Therefore, terahertz waves can detect a wealth of material information. By studying the fingerprint spectra of substances in the terahertz band, one can reveal information such as the composition of the substances and the microscopic structure of the molecules.

Terahertz spectroscopy can be divided into THz-TDS and terahertz frequency-domain spectroscopy (THz-FDS). THz-TDS is suitable for rapid broadband detection, while THz-FDS is suitable for high-resolution analysis. In terms of data form, since THz-TDS can obtain frequency-domain information through Fourier transformation, it is the most widely used. A typical THz-TDS system mainly consists of ultrafast pulse lasers, terahertz emitters and detectors, as well as time delay controllers, etc. [41]. The measurement modes include transmission mode, reflection mode and attenuated total reflection (ATR) mode. The transmission mode is often used for solid samples with small scattering and absorption, the reflection mode is suitable for solid samples with large scattering and absorption, and the ATR mode can effectively solve the problem that polar liquids are difficult to directly measure in the terahertz band [42].

### 2.2. Terahertz Metamaterial

The traditional THz-TDS system requires that the samples be prepared in powder form and sometimes subjected to high-pressure treatment to be made into tablets [43]. In substance sensing applications, THz-TDS technology still has limitations such as low sensitivity. Therefore, additional signal amplifiers specifically designed for measuring trace or liquid samples are needed.

In recent years, researchers have utilized the localized field enhancement effect of metamaterials to amplify the sample signals, effectively improving the sensitivity of terahertz technology for material detection [44]. Metamaterials are man-made periodic structures that are easy to manufacture on a large scale. The working principle of terahertz metamaterial sensors is to convert changes in the dielectric constant around the sensor into changes in the electromagnetic signal spectrum, which is manifested as changes in the position or amplitude of the resonant peak [45]. The change in refractive index is related to the dielectric constant. By using thin substrates with special metamaterial structure and low dielectric constants and low losses, the detection sensitivity of metamaterial can be enhanced. This is conducive to the detection of small changes in matter and the reduction in sample consumption. The substrate material can be rigid or flexible. In addition, the sensitivity of metamaterial terahertz sensors can also be improved by reasonably setting metamaterial and enhancing the interaction between biomolecules and terahertz electromagnetic waves. The performance of terahertz metamaterial sensors is closely related to the resonant modes they adopt [46]. Currently, the resonant modes developed in other fields include dipole resonance [47], inductive–capacitive resonance (LC resonance) [48], quasi-bound state in continuum [49], hybridization-induced transparency [50], electromagnetically-induced transparency (EIT) [51], plasmon-induced transparency [52], and Fano resonance [53].

Currently, terahertz metamaterial sensors can be categorized by material composition into several types: metal-based, semiconductor-based, carbon-based, and all-dielectric metamaterials.

Metal-based metamaterials are primarily constructed using metallic array structures. These sensors exploit localized surface plasmon resonance to achieve high detection sensitivity, in some cases reaching the single-molecule level. The metallic components themselves provide inherent advantages such as corrosion resistance and high-temperature tolerance, making them suitable for use in complex industrial environments. In addition, the design principles for these sensors—based on well-established physical mechanisms such as LC resonance and EIT—are relatively mature and extensively documented. Despite these advantages, metal-based metamaterials also present notable limitations. Their fabrication often requires high-precision techniques such as lithography and electron-beam evaporation, which are costly and resource-intensive. Moreover, once the structure is fixed, dynamically adjusting the resonance frequency is difficult, posing challenges for real-time tuning. To address this issue, phase-change materials such as vanadium dioxide (VO_2_) are sometimes incorporated, allowing for a limited degree of dynamic adjustment.

Semiconductor-based metamaterials, such as those employing indium antimonide (InSb), are notable for their dynamic control capabilities and integration potential. A key advantage of semiconductors is that their carrier concentration can be rapidly tuned through voltage or temperature, enabling dynamic switching of sensor responses. This flexibility makes them promising candidates for future applications involving chip-level integration. Nonetheless, they also face significant limitations. Many semiconductor-based materials require low-temperature environments—often achieved using liquid helium—to function effectively, which severely restricts their applicability to specialized settings. In addition, the intrinsic absorption of semiconductors introduces higher background noise, leading to a lower signal-to-noise ratio compared with metal-based metamaterials. These challenges can constrain their performance in applications where high sensitivity and signal clarity are essential.

Carbon-based terahertz metamaterial sensors, which use graphene and carbon nanotubes as core materials, offer distinct advantages due to their exceptional physical properties. Graphene, with its zero bandgap in the terahertz frequency range, provides a broadband response and exhibits high carrier mobility (up to 200,000 cm^2^/(V·s)), making it ideal for high-speed modulation. Additionally, carbon-based materials can be integrated onto flexible substrates, providing mechanical flexibility and making them suitable for wearable applications, such as real-time health monitoring. However, challenges remain in their fabrication and stability. High-quality graphene typically requires chemical vapor deposition for synthesis, and interfacial defects between graphene and the substrate can degrade sensor performance. Moreover, these materials are sensitive to environmental factors such as humidity and oxygen, requiring encapsulation for protection.

The core advantages of all-dielectric terahertz metamaterial sensors, which use materials like silicon, silica, and others as the base, lie in their high performance, accuracy, and biocompatibility. These materials exhibit extremely low loss in the terahertz frequency range, allowing for the design of high-Q resonant structures that significantly improve sensing accuracy. Additionally, their favorable biocompatibility makes them ideal for biomedical applications, such as dynamic cell monitoring. However, their limitations primarily arise from challenges in light field control and processing. The interaction between the base materials and terahertz waves is relatively weak, necessitating the design of complex, ultra-thin surface structures to enhance the light field, thereby increasing design complexity. Moreover, the fabrication of deep sub-wavelength structures requires high-precision etching, leading to higher processing costs.

## 3. Application of Terahertz Spectroscopy in Food Analysis

### 3.1. Harmful Substance Detection

From crop cultivation to consumption, food undergoes a complex series of processes, forming a lengthy processing chain. At various stages—including production, transportation, and storage—pollutants may be introduced, potentially leading to contamination and posing significant risks to human health. Food contamination arises from a wide range of sources, including pesticide residues, antibiotic residues, food additives, and mycotoxins [54,55]. Terahertz waves have been applied in biochemistry and food safety due to their advantages such as low energy, high coherence, excellent signal-to-noise ratio, and capability for non-destructive testing [56,57].

#### 3.1.1. Pesticide Residues

**Figure 3:**
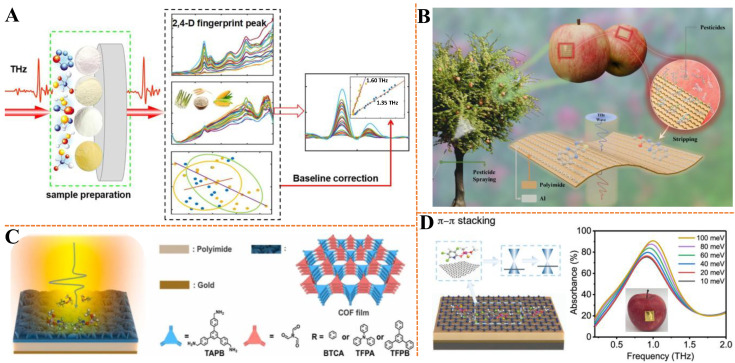
(**A**) Schematic diagram of terahertz sensing for detecting 2,4-D pesticide residues in powdered food matrices using the tablet-pressing method. Adapted with permission from [60]. Copyright 2021 Elsevier. (**B**) Schematic diagram of the pesticide biosensing principle based on flexible USM. Adapted with permission from [63]. Copyright 2025 Optica Publishing Group. (**C**) Schematic figure of terahertz absorber based on COF. Adapted with permission from [68]. Copyright 2022 Elsevier. (**D**) Schematic diagram of the principle of a graphene-based terahertz sensor for detecting chlorpyrifos methyl without using metamaterials. Adapted with permission from [69]. Copyright 2020 American Chemical Society.

In agricultural practice, pesticide residues in products are typically present at trace levels, making them undetectable through direct terahertz spectral analysis. Therefore, it is essential to integrate chemometric techniques to enable both qualitative discrimination and quantitative assessment of products with pesticide residues versus safe ones. The integration of chemometrics with terahertz spectroscopy significantly broadens the application potential of this technology [58,59]. In the case of powder samples, pesticide residues can be effectively detected using the traditional tablet-pressing method. As shown in Figure 3A, Qu et al. aimed to characterize the terahertz fingerprint peaks of 2,4-dichlorophenoxyacetic acid (2,4-D) and improve its detection in food matrices using THz-TDS [60]. Density functional theory (DFT) was employed to simulate molecular vibrations, identifying characteristic peaks at 1.35, 1.60, 2.37, and 3.00 THz. Four baseline correction methods—asymmetric least squares smoothing, adaptive iteratively reweighted penalized least squares, background correction, baseline estimation and denoising with sparsity—were compared to mitigate spectral baseline drift caused by scattering in food matrices (zizania latifolia, rice, maize). Baseline correction significantly improved detection accuracy, with the 1.35 THz peak exhibiting the best linearity and reducing the limit of detection (LOD) from 7% to 1%. These findings demonstrate that THz-TDS, when combined with effective baseline correction, enables precise quantitative analysis of 2,4-D residues, offering a robust approach for food safety monitoring.

In addition to chemometric approaches for extracting meaningful terahertz spectral features, the development of sensing methods based on terahertz metamaterials also contributes to the sensitive detection of trace pesticide residues [61]. For example, Dai et al. developed a metal–graphene hybrid metasensor for ultra-sensitive chlorothalonil detection at the pictogram-per-milliliter level, utilizing graphene’s tunable Fermi level and toroidal dipole resonance effects [62]. The sensor exhibited distinct modulation in terahertz reflection intensity with increasing pesticide concentration, achieving a LOD of 100 pg/mL. Theoretical simulations confirmed that the enhanced light–matter interaction stemmed from π–π stacking between graphene and analyte molecules, coupled with toroidal mode excitation that minimized radiative losses. This study establishes a simple yet highly sensitive platform for trace pesticide detection, with strong potential for applications in environmental monitoring and food safety. As illustrated in Figure 3B, Li et al. proposed a flexible umbrella-shaped metamaterial (USM) biosensor integrated with triple-resonance sensing based on high-order modes and a two-stage cascade ensemble learning framework for simultaneous qualitative and quantitative analysis of six pesticides (2,4-D, carbendazim, thiophanate-methyl, 2-(4-thiazolyl)benzimidazole, triazolo-pyrimidine, and tebufenozide) [63]. The biosensor design incorporated optimization of resonance depth and Q-factor to improve signal contrast, while the learning framework achieved 100% qualitative classification accuracy. For quantitative analysis of pesticide residues in the range of 0~100 μg, the system attained a root mean square error (RMSE) of 2.21 μg and a coefficient of determination (*R*^2^) of 0.9952. Moreover, the USM biosensor demonstrated excellent reusability—maintaining stable performance after 200 reuse cycles with resonance frequency shifts within 1.2%—and robust mechanical durability, showing negligible spectral variation (Δf < 5 GHz) after 1000 bending cycles. These results highlight its potential for on-site, real-time detection of pesticide residues in food safety applications.

The patterns produced by lithography and laser etching enable the realization of customizable sensing platforms. Although these metamaterials with array patterns significantly enhance the sensitivity of terahertz sensing, their processing costs are high [64,65]. Covalent organic frameworks (COFs) with high porosity and high specific surface area can be designed to have rich and characteristic interactions with target molecules, and have been widely used in the field of sensing [66,67]. In the pursuit of efficient molecular detection in the terahertz band, Xu et al. introduced two-dimensional COFs as lossy layers for terahertz absorbers, addressing the limitations of conventional materials such as low selectivity and high fabrication cost [68]. By in-situ wet-chemistry growth, COF nanofilms with hierarchical structures (specific surface areas: 736~971 m^2^/g, nanopore sizes: 1.6~2.1 nm) were directly deposited on polyimide (PI) dielectric layers, forming trilayer absorbers with a gold back-reflector (Figure 3C). Iodine doping enhanced carrier density, improving terahertz resonance. The resulting sensors demonstrated sensitive and selective detection of organophosphorus pesticides, achieving a LOD of 2.2 ng, with a 2.7~7.8 times higher response to pesticides than interferents like saccharides and antibiotics. The flexible sensors maintained performance over 1000 bending cycles and enabled practical detection of pesticide residues on apple surfaces. This work highlights the potential of reticular COF nanofilms for high-performance terahertz sensing, offering designable structures and cost-effective fabrication for rapid molecular recognition. To overcome the challenges of complex fabrication and poor bio-interface contact in metamaterial-based terahertz sensors, Xu et al. developed a metamaterial-free, flexible graphene-enabled terahertz sensor for pesticide detection [69] (Figure 3D). The sensor utilized a trilayer structure of graphene, PI, and gold, where external molecules interacted with graphene’s π electrons to modulate the Fermi level and terahertz absorption. Experimental results showed sensitive detection of chlorpyrifos methyl with a low LOD of 0.13 mg/L, and the sensor successfully identified 0.60 mg/L of pesticide on apple surfaces. The flexible design exhibited high stability, maintaining performance after 1000 bending cycles, with a fabrication cost below $2 per 10 mm^2^. This work presents a versatile, low-cost strategy for terahertz sensing at bio-interfaces, leveraging graphene’s unique electronic properties to enable label-free, non-destructive detection of molecules with π-electron structures, promising applications in agricultural and biomedical fields.

#### 3.1.2. Antibiotics

Antibiotics are a class of secondary metabolites or synthetic analogues produced by microorganisms or higher animals and plants during their life processes, which have the ability to combat pathogenic microorganisms or other active substances [70]. They can help humans and animals resist bacterial infections or infections caused by other pathogenic microorganisms [71]. As an antibacterial drug, it provides protection for the health of both humans and animals. However, excessive use of antibiotic drugs can lead to the residue of antibiotics in the bodies of humans and animals, increasing the risk of the emergence of drug-resistant bacteria, and also causing pollution of the environment as the antibiotics are excreted by humans and animals and enter the environment [72]. Effective qualitative and quantitative detection and control of antibiotics are of great significance and practical value in the fields of medicine, animal husbandry and food [73,74].

**Figure 4:**
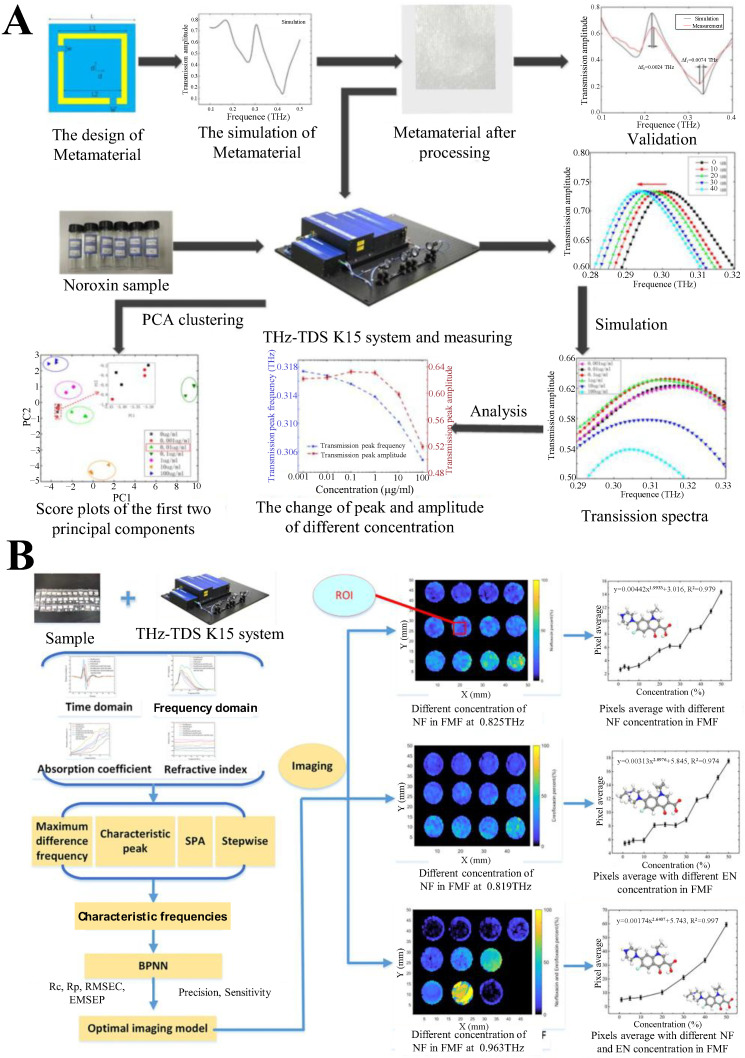
(**A**) Schematic diagram of low concentration noroxin detection experimental process. Adapted with permission from [75]. Copyright 2021 Elsevier. (**B**) Schematic diagram of visual and quantitative study of FQs in FMF. Adapted with permission from [78]. Copyright 2022 Elsevier.

To address the challenge of detecting low-concentration noroxin, a quinolone antibiotic, Li et al. designed a novel metamaterial structure and integrated it with terahertz spectroscopy [75] (Figure 4A). The metamaterial, featuring a square annular hole array on a silicon dioxide substrate, exhibited strong electromagnetic field enhancement, enabling sensitive detection of dielectric changes caused by noroxin thin films. By simulating transmission spectra of photoresists with varying thicknesses and measuring terahertz responses of noroxin solutions (0.001~100 mg/mL), the study revealed a linear relationship between noroxin concentration and the redshift of the metamaterial’s transmission peak in the terahertz band. Principal component analysis (PCA) further validated the method’s ability to distinguish low concentrations, achieving a LOD of 0.01 mg/mL—meeting China’s national standard for quinolone residues in food. This work demonstrates that terahertz spectroscopy combined with metamaterials offers a non-destructive, high-sensitivity approach for quantifying antibiotic residues, providing a foundational framework for food safety applications.

Due to the broad-spectrum effect of large-ring antibiotics, along with relatively minor side effects and the absence of teratogenicity, they have been proven to be valuable alternatives to penicillin and cephalosporins in the treatment of various infections [76]. Liu et al. investigated the use of a one-step transfer graphene-metamaterial absorber for characterizing concentrations of macrocyclic antibiotics (erythromycin, midecamycin, josamycin) [77]. The heterostructure, featuring a metal–medium–metal metamaterial with transferred graphene, exhibited distinct terahertz reflection responses to varying antibiotic concentrations. Experimental results showed that increasing antibiotic concentrations induced a blue shift in the resonant peak frequency, directly proportional to the analyte concentration. Josamycin showed the highest sensitivity, followed by midecamycin and erythromycin, attributed to differences in molecular interactions with graphene’s π-conjugated system. This study establishes a correlation between resonant peak shift and antibiotic concentration, providing evidence for using graphene-metamaterial absorbers as a versatile platform for rapid, label-free screening of macrocyclic antibiotics with high sensitivity and specificity.

**Figure 5:**
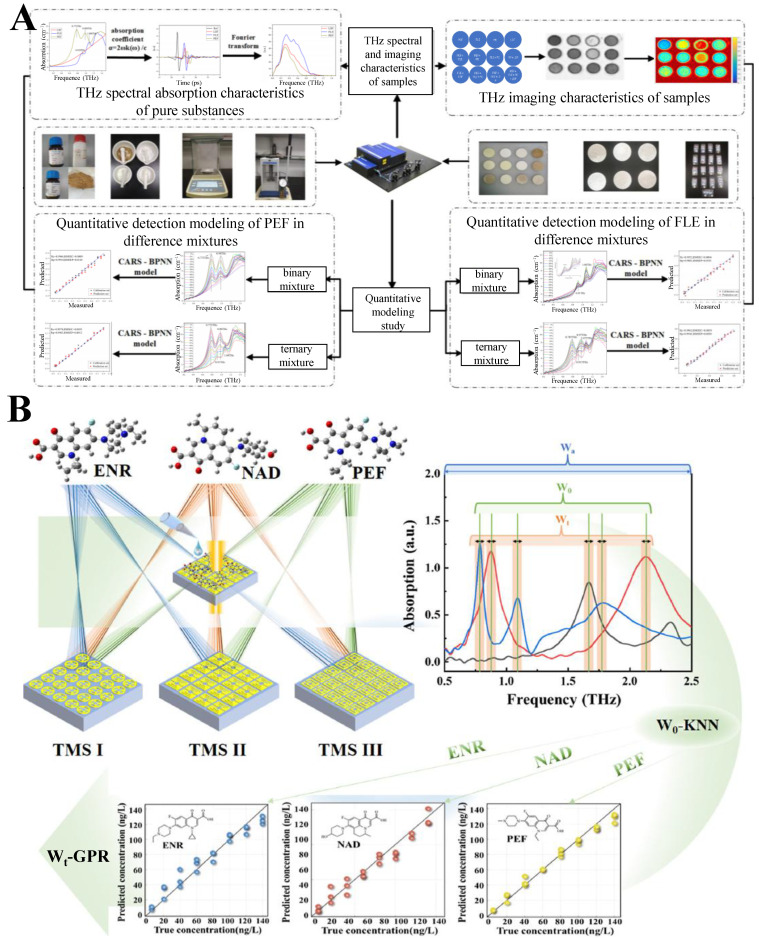
(**A**) Schematic diagram for detecting FQ antibiotics using terahertz technology. Adapted with permission from [82]. Copyright 2022 John Wiley and Sons. (**B**) Schematic diagram of qualitative and quantitative analysis of trace FQs using terahertz metamaterial sensors combined with machine learning algorithms. Adapted with permission from [83]. Copyright 2025 Elsevier.

Fluoroquinolones (FQs) are a group of highly effective synthetic chemotherapy drugs widely used in both human and veterinary medicine for the treatment of bacterial infections [79]. They have broad activity against both Gram-positive and Gram-negative bacteria [80]. The widespread use of FQs can lead to residues in animal-derived foods and enhance bacterial resistance, which poses potential risks to human health [81]. To address the challenge of detecting trace FQ antibiotics in water, Bai et al. explored the feasibility of terahertz spectral and imaging technology for visualizing and quantifying FQs in fish meal feeds (FMF) [78] (Figure 4B). Four methods—maximum frequency-domain difference, characteristic absorption peaks, successive projections algorithm (SPA), and stepwise regression—were employed to identify characteristic terahertz frequencies for norfloxacin (NF) and enrofloxacin (ENR) in binary and ternary mixtures with FMF. Terahertz images formed at selected frequencies (e.g., 0.825 THz for NF, 0.819 THz for ENR) showed distinct contrast changes with concentration. A backpropagation neural network (BPNN) was used to evaluate imaging models, revealing strong correlations between FQ concentrations and gray values in regions of interest, with determination coefficients (*R*^2^) up to 0.997. This study establishes a novel visualization method for FQs in FMF, highlighting terahertz technology’s potential for rapid, non-destructive screening of antibiotic residues in complex food matrices. As illustrated in Figure 5A, Cao et al. explored the feasibility of terahertz spectroscopy for quantifying pefloxacin (PEF) and fleroxacin (FLE) in livestock feed, leveraging characteristic absorption peaks and chemometric models [82]. PEF exhibited distinct peaks at 0.775 and 0.988 THz, while FLE showed peaks at 0.919 and 1.088 THz, enabling qualitative identification. For quantitative analysis, competitive adaptive reweighted sampling (CARS), SPA, and their combination (CARS-SPA) were used for dimensionality reduction, coupled with BPNN. The CARS-BPNN model achieved the best performance, with prediction correlation coefficients (*R*_p_) of 0.9910 for PEF in binary mixtures and 0.9963 in ternary mixtures, outperforming single-algorithm models. Ternary mixtures showed better prediction accuracy than binary ones, attributed to molecular interactions between antibiotics. This study establishes terahertz spectroscopy as a viable tool for rapid, non-destructive detection of fluoroquinolones in feed matrices, providing a foundation for practical antibiotic residue monitoring in livestock and poultry industries. As shown in Figure 5B, Zhang et al. developed a novel approach for qualitative and quantitative analysis of trace FQs by integrating resonance features of multiple terahertz metamaterials sensors (TMSs) with machine learning algorithms [83]. They designed and fabricated three TMSs with distinct patterned metal top layers, whose resonance responses varied with FQs types and concentrations. By fusing the resonance peak features of these TMSs to construct the optimal feature matrix (Wₒ), the built Wₒ-K-Nearest Neighbor (KNN) classification model achieved 100% accuracy for three FQs (ENR, nadifloxacin, PEF). Additionally, the optimal resonance peak interval feature matrix (Wₜ) was constructed by optimizing the feature width, and the Wₜ-Gaussian process regression (GPR) quantitative model with different kernel functions exhibited high accuracy, with R^2^ of 0.94~0.98 and RMSE of 6.4085~10.6540. This method enables rapid, precise, and reliable detection of trace FQs, providing a robust platform for biomolecular sensing in food safety and environmental monitoring.

Chloramphenicol (CAP) was originally derived from *Streptomyces venezuelae*. Due to its low cost, ready availability, and excellent performance in treating various infectious diseases through protein inhibition, it has been widely used in animal treatment [84,85]. However, CAP poses a potential threat to human and animal health, such as hypoplastic anemia, aplastic anemia, and thrombocytopenia [86]. Su et al. developed a sensitive and specific terahertz immunosensor based on a nanoscale gold film metamaterial (AuF-MM) for detecting CAP in milk, addressing the challenges of matrix interference and low-concentration detection [87]. The AuF-MM, functionalized with CAP–bovine serum albumin (CAP-BSA) via hydrophobic interactions, enabled competitive binding with colloidal gold-labeled CAP monoclonal antibodies (AuNPs-mAb). Changes in terahertz transmission spectra, induced by refractive index variations from immune complex formation, were used to quantify CAP residues. The sensor achieved a LOD of 5 pg/mL, with linearity (*R*^2^ = 0.99136) and minimal interference from milk matrices, showcasing superior penetration capability. Specificity tests confirmed negligible cross-reactivity with other antibiotics (ENR, amantadine, tilmicosin, florfenicol). This work presents a novel, cost-effective strategy combining terahertz metamaterials with immunocompetitive assays, offering a promising approach for trace contaminant detection in food and agricultural products.

#### 3.1.3. Additives

Food additives, as an important part of modern food industry, play a crucial role in improving the color of food, extending its shelf life, and enhancing its flavor. However, the illegal addition or excessive use of additives has become a major issue in global food safety.

To address the need for rapid and accurate detection of citrate salts (CSs) in food and pharmaceuticals, Deng et al. designed an asymmetric double-opening ring metamaterial sensor, leveraging terahertz spectroscopy to analyze low-concentration CSs solutions [88]. The sensor’s structural parameters, including opening positions and metal ring arrangement, were optimized via electromagnetic simulations, revealing that asymmetric configurations enhanced the quality factor (Q-value) and sensitivity to refractive index changes. Terahertz measurements demonstrated distinct resonant frequency redshifts for six CSs (Li-, K-, Mg-, Ca-, Fe-, and Zn-citrate) due to their varying dielectric properties, with sensitivity reaching 402 GHz/RIU. The sensor effectively differentiated CSs by correlating refractive index differences with spectral shifts, showcasing its ability to identify low-concentration analytes. This study highlights the potential of terahertz metamaterial sensors for non-destructive, high-sensitivity detection of organic salts, providing a novel approach for quality control in food and pharmaceutical industries.

Xue et al. introduced a magnetic dipole metamaterial sensor combined with an angle-scanning strategy to enhance trace food additive detection [89]. By manipulating the incident angle of terahertz waves, the sensor broadened its spectral response, covering characteristic absorption frequencies of azodicarbonamide (ADA), sorbic acid, and melamine. The all-dielectric structure, composed of lithium tantalate cylinders, exhibited a high Q-factor, enabling strong electromagnetic field localization and improved molecular interaction. Experimental results showed LODs as low as 1.65 µg/cm^2^ for ADA, 1.20 µg/cm^2^ for sorbic acid, and 1.66 µg/cm^2^ for melamine, with linear correlations between concentration and spectral shifts. This approach addresses the sensitivity limitations of traditional terahertz sensing, offering a robust platform for simultaneous identification of multiple additives in complex food matrices and paving the way for practical food safety applications.

Zhang et al. employed THz-TDS to detect vanillin and ethyl vanillin in milk powder, optimizing spectral preprocessing and chemometric models [90]. After evaluating seven preprocessing methods, the combination of multivariate scattering correction and Savitzky–Golay (SG) smoothing improved signal quality, reducing noise and baseline drift. Dimensionality reduction using CARS and SPA enhanced model efficiency, with long short-term memory (LSTM) regression models achieving the best performance: a 94.49% recognition rate for vanillin (CARS-LSTM) and 98.37% for ethyl vanillin (SPA-LSTM). These results demonstrate the feasibility of THz-TDS for non-destructive, quantitative analysis of milk powder additives, offering a rapid and accurate alternative to traditional methods for ensuring food safety compliance.

Hu et al. designed a terahertz metamaterial resonator with a resonant peak at 1.95 THz to enhance detection of benzoic acid in liquid food [91]. By integrating electromagnetic simulations and experimental measurements, the sensor’s surface dielectric sensitivity was optimized, enabling pronounced spectral shifts with varying analyte concentrations. Preprocessing via “SG + first derivative” and variable selection using CARS improved spectral quality, while a least squares support vector machine (LS-SVM) model achieved a prediction correlation coefficient (*R*_p_) of 0.9953 and a LOD of 2.36 × 10^−5^ g/mL. This study highlights the synergy between metamaterial-enhanced terahertz spectroscopy and machine learning, providing a high-sensitivity platform for trace additive analysis that meets national standard requirements and offers insights for detecting other food contaminants. Similarly, Ma et al. developed a highly sensitive terahertz metasurface sensor for detecting benzoic acid, a common food preservative, using a cross-shaped aluminum resonator integrated on a polyimide substrate [92]. The sensor was designed with a resonance dip at 0.93 THz, matching the fingerprint spectrum of benzoic acid in the terahertz range. Simulations of terahertz transmission spectra with varying thicknesses of benzoic acid revealed that both spectral frequency shifts and transmission intensities exhibited high sensitivity to trace amounts of the analyte, with the amplitude of resonance dips increasing with benzoic acid concentration. The sensor achieved a sensitivity of 0.247 THz/RIU and a figure of merit of 3.927/RIU, outperforming several previously reported sensors. This label-free, fast, and accurate detection tool shows significant potential for ensuring food safety by enabling trace analysis of benzoic acid additives.

Peng et al. investigated the terahertz properties of two food additives, sodium hexametaphosphate and sodium pyrophosphate solutions, using a cycloolefin copolymer microfluidic chip with low terahertz absorption and a THz-TDS system [93]. The microfluidic chip, featuring simple fabrication and high terahertz transmittance (>90% for 2 mm thickness), enabled measurements of solutions with concentration gradients (0.3~0.7% for sodium hexametaphosphate and 0.6~1.0% for sodium pyrophosphate). Results showed that terahertz transmission intensity increased with concentration for both solutions, attributed to anion-induced disruption of hydrogen bonds in water, reducing terahertz absorption. This study demonstrates the feasibility of combining terahertz technology with microfluidic chips for analyzing electrolyte solutions, laying a foundation for investigating ion effects on hydrogen bonds in aqueous systems.

#### 3.1.4. Pathogenic Bacteria and Toxins

Pathogenic bacteria are the main cause of foodborne diseases, posing a challenge to global public health. The common pathogenic bacteria found in food include *Staphylococcus aureus* (*S. aureus*), *Escherichia coli* (*E. coli*), *Salmonella typhimurium*, etc. [94]. Quick and accurate detection of pathogenic bacteria is a pressing issue that needs to be addressed in the fields of public health, food safety, and environmental monitoring [95].

**Figure 6:**
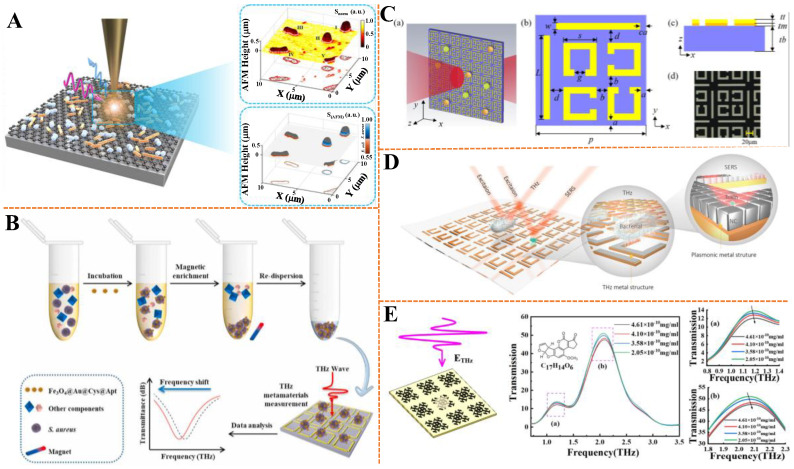
(**A**) Schematic diagram of single-bacterial identification based on terahertz near-field imaging. Adapted with permission from [96]. Copyright 2025 American Chemical Society. (**B**) Schematic diagram of terahertz sensing for *S. aureus* detection assisted by magnetic nanomaterials. Adapted with permission from [97]. Copyright 2024 Elsevier. (**C**) Schematic diagrams of the (a) sensing and unit structures ((b) top view, (c) side view, and (d) microscopic image) for the detection of two *Staphylococcus* species. Adapted with permission from [34]. Copyright 2025 Optica Publishing Group. (**D**) Schematic diagram of the hierarchical metamaterial sensor for the detection of *Pseudomonas aeruginosa* and its pyocyanin. Adapted with permission from [98]. Copyright 2021 John Wiley and Sons. (**E**) Schematic diagram of the terahertz metamaterial sensor with “X” compound double-peak structure for the detection of AFB2. (a), (b) respectively represent the locally magnified terahertz transmission images at 1.2 and 2.0 THz. Adapted with permission from [102]. Copyright 2023 Elsevier.

Zhou et al. developed a single-bacterium terahertz dielectric nanoimaging (STDN) strategy using a customized terahertz scattering-type scanning near-field optical microscopy platform [96] (Figure 6A). This strategy synchronously tracked bacterial (*S. aureus*, *E. coli*) intrinsic dielectric properties and extrinsic morphology, with its terahertz nanoimages validated by theoretical modeling and near-field measurements. Combined with a trained classifier, it achieved 99.3% accuracy in species identification and 91.6% accuracy in antibiotic susceptibility testing within 2 h, featuring label-free and culture-free detection. This proof-of-concept STDN strategy shows promise in facilitating precise bacterial infection management and counteracting antibiotic resistance. As exhibited in Figure 6B, Yu et al. developed a terahertz metamaterial biosensor based on aptamer-functionalized Fe_3_O_4_@Au nanocomposites for rapid and sensitive detection of *S. aureus* [97]. The nanocomposites Fe_3_O_4_@Au@Cys@Apt integrated magnetism for target enrichment and high refractive index in the terahertz range for signal amplification, enabling culture-free and extraction-free detection. The biosensor showed a linear relationship between resonance frequency shifts and *S. aureus* concentrations ranging from 1 × 10^3^ to 1 × 10^7^ CFU/mL, with a LOD of 4.78 × 10^2^ CFU/mL. As shown in Figure 6C, Zhong et al. presented a label-free terahertz metasurface sensing technology for simultaneous quantification and speciation of *S. aureus* and *Staphylococcus epidermidis* (*S. epidermidis*) [34]. The tailored metasurface, composed of four open resonant rings and perpendicular metal bars, enhanced terahertz wave–bacteria interaction, establishing linear correlations between resonance frequency shifts and bacterial fluid dosage. Distinct linear regression slopes (*S. aureus*: 105.90 GHz/µL, *S. epidermidis*: 45.14 GHz/µL) enabled species differentiation, with high experimental sensitivities of 556 GHz/cell μm^−2^ (*S. aureus*) and 237 GHz/cell μm^−2^ (*S. epidermidis*). This technology eliminates complex surface functionalization, offering a rapid, cost-effective solution for clinical diagnostics and food safety monitoring, particularly in resource-limited settings.

Furthermore, terahertz technology can be combined with other spectroscopic techniques to obtain more comprehensive information about pollutants. For example, Wang et al. developed cross-wavelength hierarchical metamaterials integrating microscale U-shaped terahertz resonators and nanoscale Ag nanocubes (AgNCs) superlattices for simultaneous trans-scale molecule detection [98] (Figure 6D). The microscale structure enabled terahertz sensing of micrometer-scale *Pseudomonas aeruginosa* with a LOD of 5000 cfu/mL, while the nanoscale AgNCs provided SERS enhancement for sub-nanoscale metabolites (pyocyanin) with a LOD of 20 nM. This dual-functional metamaterial was applied in smart sensory packaging, realizing real-time monitoring of pathogenic bacterial growth and metabolite production in food without opening the package, demonstrating its potential for multiscale functional detection.

Agricultural products, especially grains, are prone to contamination by mycotoxins, among which aflatoxin poses the greatest threat. Consuming food contaminated with aflatoxins can lead to serious diseases in humans [99]. The accumulation of aflatoxins in the body can cause both acute and chronic toxicity, resulting in acute liver damage, liver cirrhosis, tumors and teratogenic effects [100,101]. Hu et al. designed a terahertz metamaterial sensor with an “X” composite double-peak structure for highly sensitive quantitative detection of aflatoxin B2 (AFB2) solution [102] (Figure 6E). They observed that the amplitude of transmission peaks around 1.2 THz and 2.0 THz decreased gradually, while the peak frequency shifted to higher frequencies with increasing AFB2 concentration. A strategy of classifying concentration intervals and establishing grouped quantitative models was proposed, significantly improving the LOD for low- and medium-concentration sub-models, with the optimal grouped model achieving LODs of 7.28 × 10^−11^ mg/mL, 4.19 × 10^−9^ mg/mL, and 1.22 × 10^−7^ mg/mL for low, medium, and high concentrations, respectively. This study verified the feasibility of combining the “X” structure metamaterial with THz-TDS technology for rapid, non-destructive, and highly sensitive AFB2 detection in food. Ochratoxin A (OTA) is a type of mycotoxin produced by Aspergillus and Penicillium genera, which has harmful effects such as genetic toxicity, immunotoxicity, neurotoxicity, and hepatotoxicity [103]. Chen et al. achieved rapid determination of OTA in black tea using a terahertz ultrasensitive biosensor combining surface plasmon resonance with terahertz spectroscopy [104]. For OTA in acetonitrile solution, the detection range was 0~20 pg/µL with a LOD of 1 pg/µL. When applied to black tea, the LOD reached 1 pg/mg, which was 500 times higher than that of UV spectrophotometry and fully met European Union regulations. This method offers a simple, rapid, and low-cost approach for trace OTA quantification in foods, providing a new strategy for small molecule detection.

### 3.2. Component Analysis

Terahertz waves are highly sensitive to the vibrations of water molecules. Therefore, they can be used to detect the moisture content in grains, leaves and dairy products, and also to monitor the drying process [36]. In addition, during the drying process, the water distribution of the material will be transferred and changed, and the terahertz imaging technology provides a new way to present the water distribution [105]. In situ real-time monitoring of moisture content (MC) during fluidized bed drying is critical for process control, yet remains challenging. For example, Ren et al. integrated a whisk–broom THz-TDS imaging system with a pulsed fluidized bed dryer (PFBD) to monitor MC reduction in particulate foods like peppercorn and sweetcorn [106]. By combining THz-TDS with convolutional neural networks (CNN) and LSTM neural networks, the study established robust prediction models for MC reduction under varying drying temperatures and flow patterns. The LSTM-CNN model demonstrated superior performance on mixed datasets, achieving a coefficient of determination (*R*_cv_^2^) of 0.91 and a low RMSE of 0.03, outperforming traditional machine learning methods like partial least squares regression (PLSR) and support vector regression (SVR). This innovative approach enables non-destructive, real-time MC monitoring, addressing the limitations of conventional techniques. The successful integration of THz-TDS imaging with deep learning provides a promising strategy for enhancing process control in food drying, with implications for optimizing energy efficiency and product quality in agricultural and food industries. Hyperspectral imaging (HSI) is a powerful vibrational spectroscopy technique that integrates the advantages of machine vision and visible infrared spectroscopy to obtain spatial and spectral information from objects [107], and is also capable of visualizing water migration from time-series hyperspectral images [108]. Investigating nonthermal pretreatments on microwave vacuum-dried beef, Ren et al. combined THz-TDS and near-infrared hyperspectral imaging (NIR-HSI) to assess moisture, color, and shrinkage [109]. Osmotic pretreatment improved drying rates and color retention, while THz-TDS and NIR-HSI models showed *R*^2^ up to 0.9817 and 0.9563 for moisture loss prediction, respectively. NIR-HSI visualized reduced moisture heterogeneity after ultrasound pretreatment, highlighting the synergy of multi-spectral techniques for industrial drying optimization.

**Figure 7:**
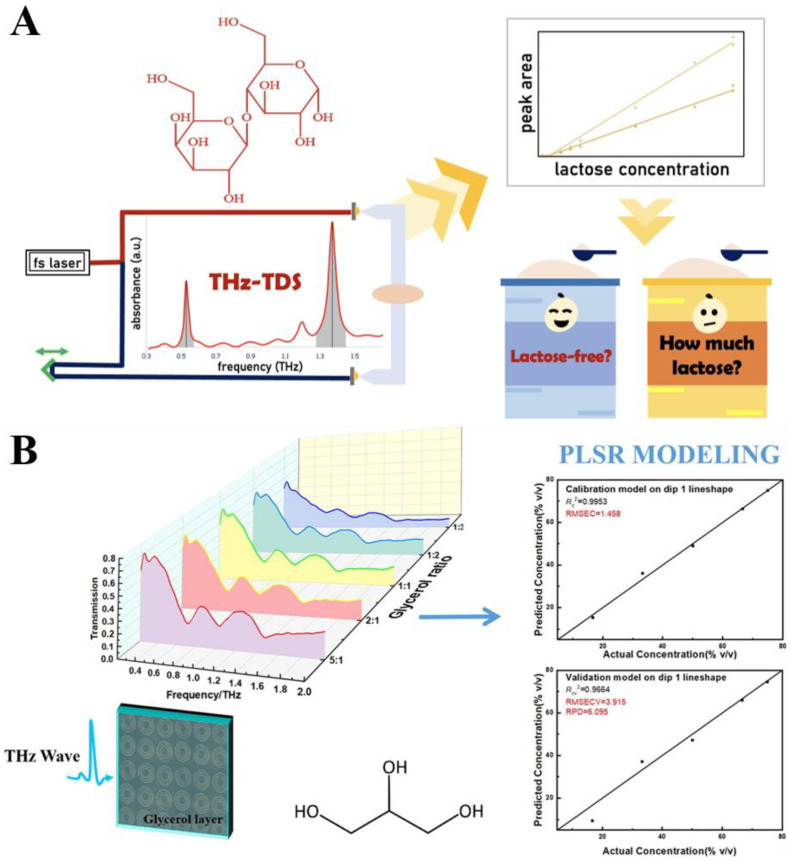
(**A**) Schematic diagram of lactose content analysis based on THz-TDS technology. Adapted with permission from [114]. Copyright 2022 MDPI. (**B**) Schematic diagram of terahertz metamaterial biosensor for detecting glycerol content. Adapted with permission from [117]. Copyright 2022 Elsevier.

The most intuitive application of terahertz spectroscopy is to detect moisture content, and in addition, terahertz spectroscopy can also reflect changes in the content of certain organic compounds in food. The gluten protein in wheat flour provides an important function for forming a sticky network with a gelatinous consistency, which makes the dough elastic and has important implications for foods such as bread and noodles [110,111]. Li et al. pioneered the use of terahertz spectroscopy for gluten detection in potato starch mixtures, comparing Gaussian process regression (GPR) and support vector machine (SVM) [112]. The GPR model achieved the best performance (*R*^2^ = 0.859, RMSE = 0.070) across a concentration range of 1.3~100%, outperforming SVM. This study demonstrates the potential of THz-TDS for non-destructive gluten quantification, offering a novel technique for ensuring gluten-free food safety and quality control. Lactose intolerance is a common condition in people with lactase deficiency and can cause symptoms such as flatulence and diarrhea [113]. Lactose is a key component in infant formula, and its accurate quantification is crucial for quality control and nutritional labeling. As shown in Figure 7A, Datta et al. employed THz-TDS to analyze α-lactose monohydrate in food samples, investigating both absorption and absorption coefficient spectra [114]. Regression models were developed using the peak area and height of characteristic absorption peaks at 0.53 THz and 1.37 THz. For pure lactose standards, the models showed satisfactory prediction performance, with the peak area of the 0.53 THz peak achieving an *R*^2^ of 0.9923 and a low RMSE. However, when applied to commercial infant formula samples, the predictions were less accurate due to baseline shifts from the formula matrix and potential lactose anomer transformations. While THz-TDS effectively verified lactose-free claims, its sensitivity to different lactose forms highlighted challenges in quantifying complex matrices. This study demonstrates the potential of THz-TDS for rapid lactose analysis in ideal conditions, underscoring the need for improved models to address real-world sample complexities.

For the above solid samples and powder samples, terahertz spectra can be directly used in combination with chemometrics, deep learning and other technologies to analyze food components, but some components are relatively low in content, or subject to moisture interference, and the terahertz signal is weak. At this point, metamaterials need to be built to significantly enhance the response of terahertz sensing [115]. Glycerol, as an intermediate product of fat metabolism, also plays an important role in food fermentation and other fields [116]. To achieve sensitive and rapid quantification of glycerol concentration, Liang et al. designed a metamaterial with internal coupling and multimodal resonances, combined with THz-TDS and PLSR [117] (Figure 7B). The study revealed that as glycerol concentration decreased, the resonant dips in the transmission spectrum exhibited red-shifts and increased full-width at half-maximum. The PLSR model built on the lineshape features of the first dip (0.45~0.85 THz) demonstrated optimal performance with a residual prediction deviation of 6.095. This metamaterial-based approach offers a novel strategy for quantitative sensing in food, pharmaceutical, and cosmetic applications by integrating terahertz spectroscopy with chemometric modeling. Organic acids are essential to the quality of fruit such as taste, acidity, color, texture and flavor [118]. Zhou et al. developed laser-engraved free-standing terahertz metamaterials for rapid analysis of fruit acids, including D-tartaric, citric, and L-malic acids [119]. By optimizing laser direct-writing parameters, the metamaterial enabled distinguishable detection of acid concentrations as low as 0.1 mg/L, outperforming traditional acid–base titration in speed and simplicity. Numerical simulations elucidated the Fano-like and dipolar resonance mechanisms, highlighting the potential of this cost-effective, high-throughput technique for in-situ analysis of flavor components in food products. Sugar is very important in daily food intake, and the sugar content not only reflects the quality of the food, but also reflects changes in the fermentation process in fermentation engineering [120]. In general, terahertz spectroscopy lacks sensitivity to sugar solutions. Aiming to enhance sensitivity for low-sugar solutions, Ishak et al. designed a circular slot frequency-selective surface (FSS) optimized to match the molecular resonances of glucose (1.98 THz) and sucrose (1.80 THz) [121]. The FSS structure improved detection sensitivity by approximately 25% for glucose and 13% for sucrose, demonstrating enhanced terahertz–matter interaction. This work presents a high-sensitivity strategy for low-concentration sugar detection in food, expanding the application of FSS in terahertz sensing.

### 3.3. Quality Assessment

**Figure 8:**
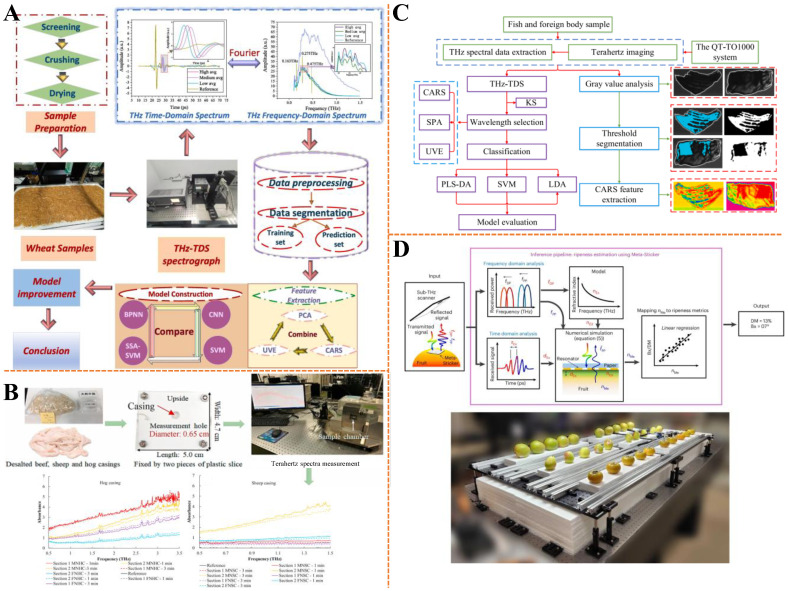
(**A**) Schematic diagram for identifying the strength of wheat gluten based on THz-TDS. Adapted with permission from [125]. Copyright 2025 Elsevier. (**B**) Main steps and spectral results of measuring sausage casing using THz-TDS (MNHC—1 min: modified natural hog casing was measured after putting in chamber for 1 min; MNHC—3 min: modified natural hog casing was measured after putting in chamber for 3 min; FNHC—1 min: fresh natural hog casing was measured after putting in chamber for 1 min; FNHC—3 min: fresh natural hog casing was measured after putting in chamber for 3 min; MNSC—1 min: modified natural sheep casing was measured after putting in chamber for 1 min; MNSC—3 min: modified natural sheep casing was measured after putting in chamber for 3 min; FNSC—1 min: fresh natural sheep casing was measured after putting in chamber for 1 min; FNSC—3 min: fresh natural sheep casing was measured after putting in chamber for 3 min). Adapted with permission from [130]. Copyright 2021 Elsevier. (**C**) Data and images processing flowchart for rapid and non-destructive detection of foreign bodies in fish based on terahertz imaging and spectroscopy. Adapted with permission from [134]. Copyright 2023 Elsevier. (**D**) Schematic diagram illustrating the principle of the sub-terahertz scanner for detecting the ripeness of fruits, and photos of actual fruits. Adapted with permission from [137]. Copyright 2025 Springer Nature.

In the food industry, the ability to monitor material changes is of great significance for controlling industrial parameters and processing progress. Terahertz spectroscopy is a non-destructive vibration spectroscopy technique that can be used to monitor food composition changes for quality control. Starch is the main source of energy in the diet, and its degradation provides most of the energy needed by humans and affects the post-meal blood sugar response [122]. There is a correlation between the digestibility rate of starch and the crystallinity, and the digestibility rate is closely related to the blood glucose index value of rice starch [123]. To investigate the potential of Fourier-transform terahertz (FT-THz) spectroscopy for monitoring crystallinity changes in rice starch after heat–moisture treatment (HMT), Guo et al. combined X-ray diffraction (XRD) and FT-THz spectroscopy to analyze A-type and Vh-type crystallinity (amylose–lipid complex) [124]. The study identified that the second derivative spectral peak at 9.0 THz was highly correlated with both crystallinity types, while additional peaks at 10.5 THz, 12.2 THz, and 13.1 THz were sensitive to Vh-type structures. Regression models showed that terahertz peaks could quantify post-HMT crystallinity changes, with strong correlations (*R*^2^ > 0.97) for both A-type and Vh-type crystallinity. This approach offers a non-destructive, efficient alternative to traditional XRD for characterizing starch crystallinity, enabling rapid assessment of structural modifications in agricultural products and facilitating insights into starch digestibility and glycemic index regulation. Wheat gluten quality is a key criterion for its application, requiring accurate and efficient discrimination. Peng et al. utilized THz-TDS combined with chemometrics and machine learning to distinguish high-, medium-, and low-gluten wheat [125] (Figure 8A). They analyzed time-domain, frequency-domain, refractive index, and absorption coefficient spectra, finding significant differences in refractive indices among different gluten types. Using CARS to select characteristic frequencies, they compared models including SVM, BPNN, improved CNN, and sparrow algorithm optimised support vector machines (SSA-SVM), with the SSA-SVM model achieving 100% discrimination accuracy. This study provides an efficient, accurate, and non-destructive method for wheat gluten strength identification, offering a theoretical basis for practical grain discrimination guided by THz-TDS.

Cocoa beans are the basis of chocolate, candy, drinks and other ingredients [126,127]. Chocolate has very little water content and a high fat content. It absorbs less terahertz radiation and has been proven to be a detectable substance in early studies [128]. The type of crystallization of the cocoa butter from the cocoa bean is crucial for tempering control in the chocolate manufacturing process. Feng et al. utilized terahertz spectroscopy to characterize three cocoa butter polymorphs (α, β′(III), β(V)) and their formation under controlled tempering conditions [129]. XRD confirmed the polymorphic forms, while terahertz spectra revealed distinct features: a sharp peak at 6.80 THz in β(V) and the original sample, and a broader peak in the α type. The study demonstrated that terahertz spectroscopy can distinguish crystallinity based on spectral intensity and peak shape variations, with β(V) showing unique spectral signatures due to its stable crystal structure. This innovative method enables non-invasive monitoring of cocoa butter crystallization during chocolate manufacturing, providing a rapid tool for quality control to ensure desirable polymorph formation (e.g., β(V) for gloss and bloom resistance) and optimize tempering processes.

Natural casings are crucial for sausage production, and their modified properties need efficient evaluation. As shown in Figure 8B, Feng et al. employed THz-TDS in both transmission and ATR modes to investigate the spectral changes in hog and sheep sausage casings before and after modification with a surfactant solution (soy lecithin and soy oil) and lactic acid [130]. They found that the terahertz absorbance of modified casings was significantly higher than that of unmodified ones, and monitored the drying process of natural hog casings, which took 31.62 min to complete under nitrogen gas flow. The results demonstrated that THz-TDS can effectively determine parameters such as water content, swelling ratio, thickness, and drying time of casings, highlighting its potential for evaluating properties of high-water-content foodstuffs like sausage casings during processing.

At present, X-ray imaging technology can identify hard foreign bodies, such as metals and high-density plastics, has been widely used in the quality inspection of agricultural products. However, low-density foreign substances remain a challenge for food quality and safety assessment. Earlier studies have demonstrated that terahertz imaging technology has the potential to detect foreign objects and map steam concentration [131]. Terahertz imaging technology can be used as an alternative tool for X-ray imaging by generating high-resolution images of the inside of food samples [132]. Sun et al. integrated THz-TDS with an electromagnetic vibration feeder to detect low-density foreign bodies (tea stalks, insects) in tea products [133]. Using K-nearest neighbor models on time-domain signals, the system achieved 100% precision, 95.6% accuracy, and 98.7% recall, outperforming absorption coefficient-based methods. THz-TDS images, processed via Otsu threshold segmentation, revealed distinct geometric features (e.g., length–width ratio, hue) to differentiate foreign bodies from tea leaves. The electromagnetic feeder mitigated signal interference from overlapping samples, enhancing detection reliability. This non-destructive approach addresses a critical gap in food safety by enabling efficient screening of low-density contaminants, applicable to industrial quality control. Similarly, Hu et al. explored the feasibility of terahertz imaging and spectroscopy for detecting endogenous (fish bones) and exogenous (metal, plastic, wooden toothpicks) foreign bodies in fish [134] (Figure 8C). They extracted spectral features using algorithms like CARS, uninformative variable elimination (UVE), and SPA, and established qualitative discriminant models including partial least squares discriminant analysis, linear discriminant analysis, and SVM. The optimal SVM model after CARS feature extraction achieved an accuracy of 99.56%, and terahertz images at 0.1~4.0 THz clearly outlined the shape, size, and location of foreign bodies. This study proved that terahertz technology provides an effective method for rapid non-destructive detection of foreign bodies in fish, contributing to food process monitoring.

Fruit ripeness plays an important role in fruit preservation and product control. Many non-destructive spectral techniques can evaluate fruit ripeness [135,136], and there is potential to develop a widely accessible, non-invasive technology for assessing fruit quality/ripeness that will benefit both the food industry and consumers. A very interesting study shows the unique contribution of terahertz spectroscopy to this aspect. As exhibited in Figure 8D, Karmakar et al. developed a sub-terahertz metamaterial sticker (Meta-Sticker) to non-invasively assess fruit ripeness by leveraging two resonant mechanisms: localized dipole resonance (sensitive to exocarp refractive index) and propagating plasmon resonance (penetrating mesocarp for inner quality analysis) [137]. By analyzing spectral shifts in these resonances, the system accurately predicted ripeness metrics (Brix, dry matter) with a cumulative normalized root mean square error of 0.54%. The Meta-Sticker, attached to fruits as a passive sensor, captures dielectric changes in both fruit layers, enabling differentiation between unripe, ripe, and overripe states. This low-cost, biodegradable technology offers a non-destructive solution for real-time ripeness assessment in distribution chains, addressing global food waste by optimizing harvest and storage decisions.

### 3.4. Adulteration Identification

Food adulteration refers to the intentional addition of harmful or cheap other ingredients in food to increase the weight or volume of food, thereby reducing costs and profiteering, resulting in changes in the nutritional composition of food and a reduction in nutritional value. Food adulteration deceives consumers, causes property damage, endangers consumers’ health and, in serious cases, causes disease and even death. Therefore, rapid, reliable and non-destructive testing of adulterated objects in food is of great significance for ensuring food safety, safeguarding consumer interests and promoting national economic development.

**Figure 9:**
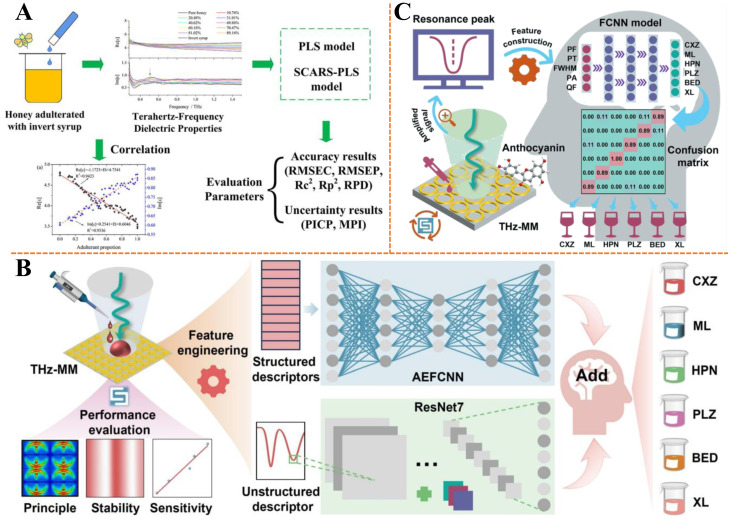
(**A**) Schematic diagram of the sensing method for identifying adulterated honey based on the dielectric properties in the terahertz region. Adapted with permission from [139]. Copyright 2022 Elsevier. (**B**) The process schematic of the dual-band terahertz metamaterial sensor combined with a deep learning model fusing 1D and 2D descriptors for synergistic identification of red wine varieties. Adapted with permission from [148]. Copyright 2025 Elsevier. (**C**) Schematic of the deep learning-driven terahertz metamaterial sensor for distinguishing different red wines. Adapted with permission from [149]. Copyright 2025 Elsevier.

Honey is rich in carbohydrates, amino acids, organic acids, vitamins, minerals, etc. [138]. Its special nutritional properties and limited supply make it an easy target for adulteration. Low-cost sugar and commercial syrups are common substances in honey adulteration. To address the need for rapid and non-destructive detection of invert syrup adulteration in acacia honey, Liu et al. utilized terahertz spectroscopy to analyze dielectric properties (real and imaginary parts of the complex dielectric constant) across 0.3~1.5 THz [139] (Figure 9A). By applying the stability competitive adaptive reweighted sampling (SCARS) algorithm to select effective variables and constructing PLSR models, the study demonstrated that the SCARS-PLSR model combining both dielectric components achieved optimal performance, with high determination coefficients (*R*_p_^2^ > 0.97) and low prediction errors. This approach highlights the potential of terahertz dielectric spectroscopy combined with chemometrics to distinguish adulterated honey, offering a robust strategy for authenticity verification in the food industry.

Ginseng is an excellent healthcare herb with high edible value and medicinal value [140]. Due to its high economic value, adulteration is easy to achieve. The effects and prices of *Panax quinquefolius* (American ginseng) from different origins are obviously different, so it is of great significance to distinguish the source of *Panax quinquefolius*. Liu et al. proposed a rapid and accurate method for identifying the origin of *Panax quinquefolius* using terahertz spectroscopy and random forest (RF) algorithm [141]. Extinction coefficients in the 0.2~1.3 THz range were first processed via PCA to reduce dimensionality and noise, followed by parameter optimization using a genetic algorithm. The optimized RF model achieved an identification accuracy of 90%, with training and validation sets achieving 100% and 92.5% accuracy, respectively. While samples from Canada and America showed slight misjudgments due to geographical similarity, the method effectively distinguished Chinese-origin ginseng with 100% accuracy. This study demonstrates the feasibility of terahertz spectroscopy combined with machine learning for non-destructive origin tracing, offering a valuable tool for ginseng quality control and market authenticity assessment. In addition to ginseng origin being easy to adulterate, its components are also easy to mix into other low-economic-value components. Pan et al. developed a quantitative method for detecting sucrose adulteration in red ginseng using THz-TDS combined with Monte Carlo uninformative variable elimination (MCUVE) and SVR [142]. By extracting absorption spectral features and leveraging the nonlinear fitting capability of SVR, the proposed MCUVE-SVR model achieved a high correlation coefficient (*R*^2^ > 0.99) and low RMSE (<1.2%), outperforming linear PLSR models. Notoginseng is usually sold in powder form, so it is difficult for consumers to distinguish the original grade from its appearance [143]. In order to earn high profits, many illegal traders sell inferior products as good products, which not only damages the interests of consumers, but also affects the market rules. Li et al. utilized THz-TDS to detect adulteration in Panax notoginseng powder with zedoary turmeric, wheat, or rice flour, combining UVE, SPA with BPNN, LS-SVM, and partial least squares (PLS) [144]. The UVE-BPNN model achieved 95% classification accuracy in qualitative analysis, outperforming SPA-BPNN (92.5%). For quantitative analysis, LS-SVM with a radial basis function kernel yielded superior results for wheat and rice flour adulteration. The prediction correlation coefficients were 0.93 and 0.934, respectively, while PLS performed best for rice flour (*R*_p_ = 0.942).

Lin et al. investigated the identification and quantification of adulterated collagen powder using terahertz spectroscopy, comparing spectral characteristics (power, absorption coefficient, refractive index, transmittance) and chemometric models [145]. The Gaussian filter–genetic algorithm–linear discriminant analysis model achieved 96.96% accuracy in classification, while the genetic algorithm–partial least squares regression model demonstrated excellent quantitative performance (*R*_p_^2^ = 0.93~0.99) for different adulterants (plant protein, corn starch, wheat flour). This work highlights the critical role of spectral feature selection and model optimization in enhancing terahertz-based adulteration detection, offering a versatile framework for food safety monitoring.

Red wine is considered a functional drink and usually contains a variety of beneficial chemicals such as acids, alcohols, trace elements, amino acids, phenols and inorganic salts, which give red wine antioxidant, anti-inflammatory, anti-cancer and anti-aging properties, among others [146,147]. It is not uncommon for low-value red wines to be labeled as high-value red wines on the market, requiring rapid identification of red wine varieties. Yu et al. developed a dual-band terahertz metamaterial sensor composed of a polyimide dielectric layer and a gold resonant layer (circle and cross structure) [148] (Figure 9B). Structural parameters were optimized to achieve optimal performance (substrate thickness: 18 μm, metal thickness: 0.2 μm, ring width: 6 μm, cross length: 61 μm, cross width: 1.8 μm), with evaluated stability and sensitivity. Using the weight factor method, the sensor accurately predicted anthocyanin (*R*^2^ = 0.9989) and tannin (*R*^2^ = 0.9916) concentrations. Ten structural descriptors and one non-structural descriptor were constructed, and a fusion model combining AEFCNN and ResNet7 in Add mode achieved excellent red wine variety identification performance (precision: 0.9848, recall: 0.9833, F1-score: 0.9833, accuracy: 0.9833). This work demonstrates the feasibility of terahertz metamaterial combined with deep learning for quality detection of complex liquid foods, providing a novel technology for red wine anti-counterfeiting. In addition, as shown in Figure 9C, Yu et al. designed a terahertz metamaterial sensor with a circular gold structure on a polyimide substrate, combined with deep learning to distinguish red wine varieties [149]. After optimizing structural parameters (substrate thickness, metal thickness, ring width) and constructing resonance peak descriptors (peak frequency, transmittance, full width at half-maximum), a fully connected neural network (FCNN) model achieved high accuracy (90.74%), precision (91.11%), and recall (90.74%). This study demonstrates the synergy between terahertz metamaterial signal enhancement and deep learning for extracting subtle spectral features, paving the way for advanced non-destructive detection in the food and beverage industry.

## 4. Summary and Prospects

Terahertz spectroscopy, with its unique properties in the 0.1~10 THz frequency range, offers several key advantages. First, it is highly sensitive to low-energy molecular motions—such as the rotation of polar molecules, vibration of crystal phonons, and hydrogen bonding—enabling the creation of material-specific “molecular fingerprints” [115]. These fingerprints can assist in drug counterfeiting detection, explosive identification, and enantiomer analysis. Second, as a non-ionizing radiation, terahertz spectroscopy can be safely used in non-destructive testing of living cells, tissues, and food, without the risk of DNA damage [65]. Third, it can penetrate most non-metallic and non-polar materials, including plastics, painting coatings, and tooth enamel, enabling the detection of packaged items, imaging of artwork drafts, and early diagnosis of dental caries [25]. Additionally, its high spectral resolution supports quantitative chemical analysis and the monitoring of subtle molecular changes. Its coherence properties also meet the requirements of interferometric imaging, such as defect detection in composite materials and sub-nanometer precision thickness measurements in films. As a result, terahertz spectroscopy plays an irreplaceable role in semiconductor manufacturing, security inspection, and other fields. Terahertz spectroscopy technology can obtain optical parameters such as the refractive index, absorption coefficient, and reflection coefficient of substances by utilizing their absorption within a specific frequency range. Due to its advantages such as fingerprint property, low energy, strong penetration, and coherence, it has made significant contributions in the field of food recently. At present, its applications mainly include component analysis, quality assessment, adulteration identification, and harmful substance detection. Terahertz spectroscopy is highly sensitive to moisture and is often used for detecting moisture during the drying process of materials. In addition, other organic chemical components in the materials can also be detected by terahertz spectroscopy to observe their changes. Based on the terahertz spectroscopy, the content levels of certain or multiple chemical components in the food matrix can be further compared with the standard values, thereby enabling quality assessment and adulteration identification. In the application of harmful substance detection, for powder samples, terahertz time-domain spectroscopy can be used for analysis by means of the traditional pressing method. Usually, algorithms such as chemometrics or machine learning need to be combined. With the development of metamaterials in the terahertz band, terahertz technology has achieved higher sensitivity, enabling it to be more widely applied in the detection of organic molecules and various harmful substances.

Terahertz spectroscopy, as an emerging technological approach, has achieved certain development in the field of food, but it is still in its infancy and faces many challenges: (i) The THz-TDS system has a complex optical path and a large volume. It relies on precise femtosecond lasers and detectors, and the equipment cost is very high. When the sensitivity is insufficient, terahertz metamaterials with certain processing costs usually need to be prepared [150,151]. (ii) The design of metamaterial sensors requires precise regulation of subwavelength structures (such as split ring resonators or photonic crystals), involving electromagnetic simulation, material processing (such as electron beam lithography or chemical vapor deposition) and other complex processes, which are demanding for laboratory conditions and technical personnel, restricting the rapid promotion of technology [152]. The common processing methods for metamaterials currently include lithography and laser direct writing technology, both of which can facilitate large-scale production. However, designing the metamaterial structure in advance is necessary, contributing to relatively high processing costs. This is one of the reasons why the research on terahertz spectroscopy technology based on metamaterials in the field of food analysis has mainly remained at the laboratory stage and is difficult to be applied in small- and medium-sized food enterprises. (iii) Water is a polar molecule that strongly absorbs terahertz waves (especially in the 0.1~1 THz band), resulting in reduced detection accuracy in high humidity environments [153,154]. For instance, when analyzing the chemical composition of agricultural products, the moisture on the surface or inside the sample can mask the characteristic spectra of the target substances. This requires additional drying preprocessing or the use of algorithms that resist moisture interference (such as spectral subtraction), which will increase the complexity of the analysis [155,156]. Additionally, water vapor in the air will introduce background noise, and a nitrogen purification system or a highly sealed detection chamber is needed, which will increase the equipment size and operating costs. (iv) Traditional terahertz spectroscopy relies on molecular vibration fingerprints. However, structural isomers (such as chiral molecules) or interfering components in complex matrices can easily cause spectral line overlap. This requires specific modifications (such as antibodies, aptamers) or multi-dimensional spectroscopy fusion techniques (such as combining with Raman spectroscopy) to enhance the specificity of recognition [157]. (v) There is a lack of unified testing standards. The spectral acquisition parameters (such as scanning range and resolution) of different devices, as well as the data processing methods, vary greatly, resulting in low data comparability between different laboratories. (vi) The terahertz spectroscopy database for key substances related to the food industry is still under construction. The existing data mostly focus on a single component and lack spectral feature annotations in complex matrices, which limits the generalization ability of machine learning models.

In conclusion, the complex composition of food matrices and the high cost of terahertz spectrometers pose challenges to the practical application of terahertz spectroscopy in the food industry. As a result, most research in this field remains at the laboratory stage, particularly in areas involving terahertz metamaterial-based sensing applications. Despite this limitation, numerous studies have confirmed the strong potential of terahertz technology for non-destructive, rapid, and label-free analysis of food quality, safety, and composition. Looking ahead, advancements in terahertz source and detector technologies are expected to reduce system costs and improve performance, thereby facilitating the broader adoption and practical deployment of terahertz spectroscopy in the food industry. Future research on terahertz technology for the food industry should focus on the following aspects. (i) By integrating nanomaterials and biometric elements, a new type of metamaterial structure can be designed to enhance sensitivity and anti-interference ability. (ii) Deeply integrate machine learning algorithms to fully exploit spectral information. (iii) Develop a multimodal technology platform, integrating terahertz spectroscopy with machine vision, Raman spectroscopy, near-infrared spectroscopy, etc., to construct a multi-dimensional detection system, improving the analytical ability for complex samples. (iv) Establish and supplement a terahertz spectroscopy database for agricultural products and food components, covering the spectral characteristics of common pollutants, additives, and pathogens, and formulating unified detection procedures and data formats. (v) Develop a lightweight terahertz spectrometer, combining microfluidics and other technologies to construct a portable detection chip, integrating sample collection, spectral acquisition, and data analysis, suitable for rapid on-site detection.

Furthermore, to facilitate the practical application of terahertz spectroscopy technology in food analysis and food enterprises in the future, we propose to adopt the following three-stage approach: (1) technical understanding—gaining a comprehensive knowledge of the principles, capabilities, and limitations of terahertz spectroscopy and metamaterial sensors; (2) demand matching—identifying specific analytical needs within the food industry that align with the unique advantages of terahertz technology; and (3) implementation—integrating terahertz systems into practical workflows, considering factors such as cost, scalability, and regulatory compliance. We hope that this framework will enable readers to apply terahertz spectroscopy technology to their food analysis requirements.

(1) Technical understanding. The application of terahertz spectroscopy in food analysis necessitates a comprehensive understanding of both its advantages and limitations, as well as its applicability to various analytical contexts. To determine the specific areas where this technology can be effectively utilized, it is beneficial to consult relevant research papers, review articles, and engage with experts from related industries or research institutions. In simple terms, the advantages of terahertz spectroscopy include non-destructive detection (allowing for analysis without sample destruction, including sealed packaged foods), fingerprint-level component identification (enabling the synchronous differentiation of multiple components), rapid response (providing second-level detection, ideal for real-time quality control in production lines), and low interference characteristics (penetrating non-metallic packaging without ionizing radiation). These features make it particularly suited for the analysis of solid or semi-solid foods with consistent components. However, terahertz spectroscopy does have some limitations. For example: (i) Strong absorption of water vapor results in a low signal-to-noise ratio when analyzing foods with high moisture content (>60%). (ii) Traditional terahertz spectroscopy exhibits low sensitivity to trace substances (<0.1 mg/kg). While signal enhancement devices, such as metamaterials, can improve the detection of trace substances, their use introduces additional processing costs. Although water vapor interference is considered a limitation, it can also be advantageous for monitoring the drying process of food materials.

(2) Demand matching. Given the varying prices of terahertz time-domain spectroscopy systems, which are generally expensive, it is essential to select commercially available instruments that meet the specific requirements of food industry applications. The compatibility of these systems should be assessed based on factors such as detection targets, sample characteristics, application scenarios, and budget considerations. Terahertz spectroscopy is well-suited for applications such as the quantitative analysis of consistent components (e.g., sugar, fat), quality grading, adulteration screening, solid or sealed packaging, and in-line quality control on production lines. However, its use for long-term applications requires a medium-to-high budget. Due to limitations in instrument costs and the expense of fabricating metamaterials, terahertz spectroscopy is currently unsuitable for trace-level safety detection (e.g., low-concentration pesticide residues), rapid determination of single indicators (e.g., pH, salt content), or analysis of high-moisture liquids and highly heterogeneous samples. In these cases, traditional technologies are more economically viable and efficient. For example, online adulteration detection in edible oil production and batch screening of milk powder components in laboratories are highly compatible with terahertz spectroscopy, while trace pesticide detection in vegetables and rapid analysis of fresh milk is better suited for traditional methods. Research on terahertz spectroscopy in these applications is still largely in the laboratory stage.

(3) Implementation. When the needs of readers align with the capabilities of terahertz technology (or instruments) and sufficient financial resources are available, the practical implementation of these technologies can be further advanced. To facilitate this, the following steps are recommended: (i) Strengthen the foundation of spectral principles and food detection standards through literature, textbooks, and industry conferences. (ii) Conduct small-scale verification of technical accuracy (repeatability deviation < 1%, deviation from national standards < 2%) through collaborative research and prototype trials to mitigate the risks associated with large-scale implementation. (iii) Select equipment based on specific scenarios (e.g., laboratory desktop instruments for batch analysis, online systems integrated into production lines, and portable devices for on-site sampling), prioritizing those with food-specific configurations. (iv) Finally, develop quantitative and qualitative models (in conjunction with chemometrics) and create enterprise standard operating procedures or contribute to industry standards. Additionally, the identity of the readers may require differentiated promotion. Enterprises can focus on production pain points, testing institutions can expand their service capabilities, and research teams can break through technical bottlenecks (such as high moisture detection or artificial intelligence model optimization), and achieve technology implementation through interdisciplinary/unit collaboration.

## Data Availability

The original contributions presented in the study are included in the article; further inquiries can be directed to the corresponding author.

## Abbreviations

The following abbreviations are used in this manuscript:

THz-TDS	Terahertz frequency-domain spectroscopy
ATR	Attenuated total reflection
2,4-D	2,4-dichlorophenoxyacetic acid
DFT	Density functional theory
LOD	Limit of detection
USM	Umbrella-shaped metamaterial
RMSE	Root mean square error
COF	Covalent organic frameworks
PI	Polyimide
PCA	Principal component analysis
FQs	Fluoroquinolones
FMFs	Fish meal feeds
SPA	Successive projections algorithm
NF	Norfloxacin
ENR	Enrofloxacin
BPNN	Backpropagation neural network
PEF	Pefloxacin
FLE	Fleroxacin
CARS	Competitive adaptive reweighted sampling
TC4	Three-concentric C4-symmetrical split-ring
MRR-CSF	Multiple resonance response competition and selection fusion
KNN	K-Nearest Neighbor
CAP	Chloramphenicol
AuF-MM	Gold film metamaterial
CAP-BSA	CAP–bovine serum albumin
AuNPs-mAb	Colloidal gold-labeled CAP monoclonal antibodies
CSs	Citrate salts
ADA	Azodicarbonamide
SG	Savitzky–Golay
LSTM	Long short-term memory
LS-SVM	Least squares support vector machine
*S. aureus*	*Staphylococcus aureus*
*E. coli*	*Escherichia coli*
STDN	Single-bacterium terahertz dielectric nanoimaging
*S. epidermidis*	*Staphylococcus epidermidis*
AgNCs	Ag nanocubes
AFB2	Aflatoxin B2
MC	Moisture content
PFBD	Pulsed fluidized bed dryer
CNNs	Convolutional neural networks
PLSR	Partial least squares regression
SVR	Support vector regression
HSI	Hyperspectral imaging
NIR-HSI	Near-infrared hyperspectral imaging
GPR	Gaussian process regression
SVM	Support vector machine
FSS	Frequency-selective surface
FT-THz	Fourier-transform terahertz
HMT	Heat-moisture treatment
XRD	X-ray diffraction
SSA-SVM	Sparrow algorithm optimised support vector machines
UVE	Uninformative variable elimination
Meta-Sticker	Metamaterial sticker
SCARS	Stability competitive adaptive reweighted sampling
RF	Random forest
MCUVE	Monte Carlo uninformative variable elimination
PLS	Partial least squares
FCNN	Fully connected neural network
